# Factor V variants in bleeding and thrombosis

**DOI:** 10.1016/j.rpth.2024.102330

**Published:** 2024-01-26

**Authors:** Adarsh K. Mohapatra, Alice M. Todaro, Elisabetta Castoldi

**Affiliations:** Department of Biochemistry, CARIM, Maastricht University, Maastricht, the Netherlands

**Keywords:** bleeding, factor V, FV-short, mutation, venous thrombosis

## Abstract

A state-of-the-art lecture titled “Factor V variants in bleeding and thrombosis” was presented at the International Society on Thrombosis and Haemostasis (ISTH) congress in 2023. Blood coagulation is a finely regulated cascade of enzymatic reactions culminating in thrombin formation and fibrin deposition at the site of injury. Factor V (FV) plays a central role in this process, as its activated form is an essential procoagulant cofactor in prothrombin activation. However, other molecular forms of FV act as anticoagulant cofactors of activated protein C and tissue factor pathway inhibitor α, respectively, thereby contributing to the regulation of coagulation. This dual procoagulant and anticoagulant character makes FV a central regulator of the hemostatic balance, and quantitative and qualitative alterations of FV may be associated with an increased risk of bleeding or venous thrombosis. Here, we review the procoagulant and anticoagulant functions of FV and the manifold mechanisms by which *F5* gene mutations may affect the balance between these opposite functions and thereby predispose individuals to bleeding or venous thrombosis. In particular, we discuss our current understanding of the 3 main pathological conditions related to FV, namely FV deficiency, activated protein C resistance, and the overexpression of FV-short, a minor splicing isoform of FV with tissue factor pathway inhibitor α–dependent anticoagulant properties and an emerging role as a key regulator of the initiation of coagulation. Finally, we summarize relevant new data on this topic presented during the 2023 ISTH Congress.

## 1 Introduction

Blood coagulation is a cascade of enzymatic reactions that is initiated by the exposure of circulating blood to subendothelial tissue factor and culminates in thrombin generation and fibrin deposition at sites of vascular injury. Thrombin generation occurs in 2 phases, each regulated by a different anticoagulant system: the initiation phase, which produces the first traces of thrombin, is regulated by tissue factor pathway inhibitor (TFPIα and TFPIβ isoforms) [1], while the propagation phase is controlled by the serine protease activated protein C (APC), which is activated by thrombin bound to thrombomodulin on the endothelial cell surface [2]. TFPIα and APC share the common cofactor protein S.

Factor V (FV), a liver-derived glycoprotein present in plasma and platelet α-granules, contributes to coagulation and its regulation by serving as a non-enzymatic cofactor in both procoagulant and anticoagulant reactions [3]. These opposite functions are managed through different molecular forms that are generated primarily through cleavage of FV by various proteases but also through alternative splicing of the *F5* transcript. Our current understanding of the complex role(s) of FV in the maintenance of the hemostatic balance has been largely shaped by the study of 3 FV-related pathological conditions associated with bleeding or venous thrombosis, each illuminating a different function of FV.

A rare case of congenital **FV deficiency**, described by Paul Owren in 1947, led to the discovery of FV as a new coagulation factor with an essential function in prothrombin activation [4,5]. Purification and biochemical characterization of the new factor resulted in detailed understanding of FV activation and procoagulant activity as a non-enzymatic cofactor of activated factor X (FXa) in the prothrombinase complex [6]. Moreover, cloning and sequencing of the *F5* cDNA [7] and gene [8] paved the way for genetic studies of FV deficiency and other FV-related disorders.

The interest in FV was revived in the mid-90s by the discovery of **APC resistance** as the most common risk factor for venous thrombosis [9] and the identification of the FV Arg506Gln (FV Leiden) mutation as its main genetic cause [10]. The mechanistic studies that accompanied these findings highlighted the dual role of FV as a substrate of APC in FVa inactivation and as a cofactor for APC (and protein S) in factor VIIIa (FVIIIa) inactivation, providing the first evidence for an anticoagulant role of FV.

More recently, the elucidation of the **East Texas bleeding disorder** [11] has revealed the existence of a minor splicing isoform of FV, known as FV-short, that acts as a carrier and a cofactor of TFPIα [12]. Apart from unveiling additional anticoagulant properties of FV, this discovery has spawned several studies on the functional interactions among FV, TFPIα, and protein S, shedding new light on the regulation of coagulation initiation [13,14].

Here, we summarize the main lessons arising from these FV-related disorders. After reviewing the procoagulant and anticoagulant functions of FV, we discuss the mechanisms by which *F5* gene mutations can alter their delicate balance and thus increase the risk of bleeding or venous thrombosis.

## 2 FV Procoagulant Function and Its Regulation

**Figure 1:**
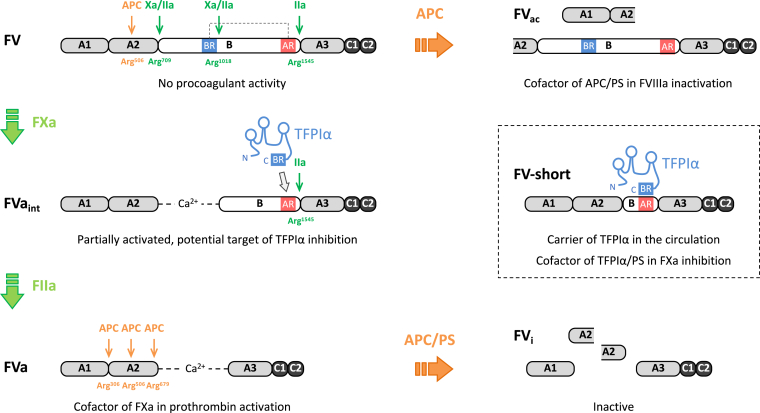
**Overview of the molecular forms of FV and their procoagulant and anticoagulant functions.** FV is secreted as a single-chain inactive precursor stabilized by an electrostatic interaction between a basic region (BR) and an acidic region (AR) within the B domain. FV cleavage by FXa and/or thrombin (FIIa) at Arg^709^, Arg^1018^, and Arg^1545^ converts FV into the prothrombinase cofactor FVa (FV activation, green arrows). This pathway generates an intermediate with an exposed AR (FVa_int_), whose prothrombinase activity and full activation can be inhibited by TFPIα. Similar forms of partially activated FV are released from activated platelets (not shown). FVa_int_ also resembles FV-short (inset), a FV splicing isoform with an exposed AR that binds TFPIα with high affinity and is complexed with it in plasma. FV-short maintains TFPIα in the circulation and enhances its anti-FXa activity in synergy with protein S. FVa is inactivated by APC and its cofactor protein S through cleavage at Arg^306^, Arg^506^ and Arg^679^ (FV inactivation, orange arrows). APC can also cleave the precursor FV at Arg^506^ (orange arrow) to generate FV anticoagulant (FV_ac_), which acts as a cofactor of APC and protein S in FVIIIa inactivation. APC, activated protein C; TFPIα, tissue factor pathway inhibitor α; PS, protein S.

Plasma FV, circulating at a concentration of 20 to 25 nM, is a single-chain inactive precursor composed of A1-A2-B-A3-C1-C2 domains. After proteolytic removal of the large and heavily glycosylated B domain, it is converted into its activated form (FVa), which consists of a heavy chain (A1-A2) and a light chain (A3-C1-C2) linked *via* a Ca^2+^-ion (Figure 1). The three-dimensional structures of FV (excluding the largely disordered B domain) and FVa have been recently solved by cryogenic electron microscopy, illustrating the spatial arrangement of the A and C domains and the conformational changes that accompany FV activation [15].

In contrast to FV, FVa has high affinity for FXa and combines with it on activated cell membranes to form the prothrombinase complex, thereby changing the biochemical pathway of FXa-catalyzed prothrombin activation [16,17] and accelerating this reaction by several orders of magnitude [6]. This extremely potent procoagulant function, that makes FV indispensable to life [18], is tightly regulated at the level of both FV activation and activity.

FV is activated through limited proteolysis at Arg^709^, Arg^1018^, and Arg^1545^ by FXa and/or thrombin [19] (Figure 1). These proteolytic events progressively remove the B domain, thereby dismantling the autoinhibitory mechanism that maintains FV in the inactive state, namely a tight electrostatic interaction between a basic region (BR, residues 963-1008) and an acidic region (AR, residues 1493-1537) within the B domain [20].

Research over the last 10 years has uncovered the existence and physiological relevance of partially activated forms of FV that lack (or have lost) the BR but retain the AR [13,14]. FV molecular species with these characteristics, collectively known as FV_AR_, circulate at very low levels in plasma (FV-short) but are also released by activated platelets (platelet FV) or generated by FXa-catalyzed cleavage of plasma FV at Arg^709^ and Arg^1018^ (FVa_int_) in the early phases of coagulation (Figure 1). FV_AR_ forms share the important property that their prothrombinase activity can be inhibited by TFPIα [21], which circulates in complex with FV-short in plasma but is also released locally by activated platelets. TFPIα is a Kunitz-type protease inhibitor that targets FVIIa and FXa [1], but its C-terminus contains a BR that is highly homologous to the BR of FV and can bind with high affinity to the exposed AR of FV_AR_ species [21]. This interaction prevents the assembly of FV_AR_ with FXa, while the Kunitz-2 domain of TFPIα blocks the FXa active site, together resulting in effective inhibition of prothrombinase activity [21,22]. In addition, TFPIα binding to the AR protects the nearby Arg^1545^ cleavage site, thereby delaying the conversion of FV_AR_ forms into fully activated FVa and maintaining them under TFPIα control [23]. These regulatory mechanisms are thought to play an important role at the onset of coagulation by controlling the transition from the initiation to the propagation phase. Responses to minor procoagulant stimuli would be extinguished by TFPIα, whereas stronger procoagulant stimuli would overcome TFPIα inhibition, leading to thrombin formation and coagulation amplification *via* feedback activation of FV and FVIII.

Having lost the AR, FVa is insensitive to TFPIα inhibition [21,23], but it can be inactivated by APC through proteolytic cleavage of the heavy chain at Arg^306^, Arg^506^, and Arg^679^ [24,25] (Figure 1). The Arg^506^ site is usually cleaved first, causing partial loss of FXa-cofactor activity, but complete FVa inactivation requires cleavage at the phospholipid-dependent Arg^306^ site, which is greatly stimulated by the APC-cofactor protein S [[26], [27], [28]]. In contrast, cleavage at Arg^679^ plays a minor role in the loss of cofactor activity [25]. Since APC-catalyzed FVa inactivation in free solution is very inefficient, most FVa is inactivated on membrane surfaces, where phospholipid composition modulates the reaction rate as well as the effect of protein S [27].

When FVa is incorporated in the prothrombinase complex, it is inactivated by APC ∼100-fold more slowly than free FVa [29]. In fact, FXa protects the Arg^506^ cleavage site by competing with APC for the same binding site on FVa [26,28], whereas prothrombin interferes with FVa cleavage at both Arg^306^ and Arg^506^ [30,31], also counteracting the stimulation by protein S [30]. Therefore, effective prothrombinase inhibition requires the concerted action of TFPIα (targeting FXa), APC (targeting FVa), and their common cofactor protein S [32].

## 3 FV Anticoagulant Functions

### 3.1 APC-cofactor activity

Early studies on the molecular bases of APC resistance revealed that, while FVa is a substrate of APC, FV stimulates the proteolytic inactivation of membrane-bound FVIIIa by the APC/protein S complex [[33], [34], [35]]. Although the exact structural requirements for the expression of this anticoagulant activity of FV are still poorly characterized, it has been shown that FV needs to be cleaved by APC at Arg^506^ in order to function as an APC-cofactor [36] (Figure 1). Moreover, the APC-cofactor activity is irreversibly lost after complete FV activation through cleavage at Arg^1545^ [37]. The physiological relevance of the APC-cofactor activity of FV is underscored by the risk of venous thrombosis associated with APC-resistant FV variants that lack this activity (see below).

### 3.2 TFPIα-cofactor activity

In recent years, evidence has accumulated that FV also exerts anticoagulant effects by interacting with TFPIα. Based on the observations that plasma TFPIα levels correlate with plasma FV levels and that FV immunodepletion removes TFPIα from plasma, it was proposed that FV and TFPIα circulate as a complex and that this complex protects TFPIα from truncation and/or clearance [38]. Moreover, it was shown that FV synergizes with the TFPIα-cofactor protein S [39] to enhance the TFPIα-mediated inhibition of membrane-bound FXa [[40], [41], [42], [43]].

**Figure 2:**
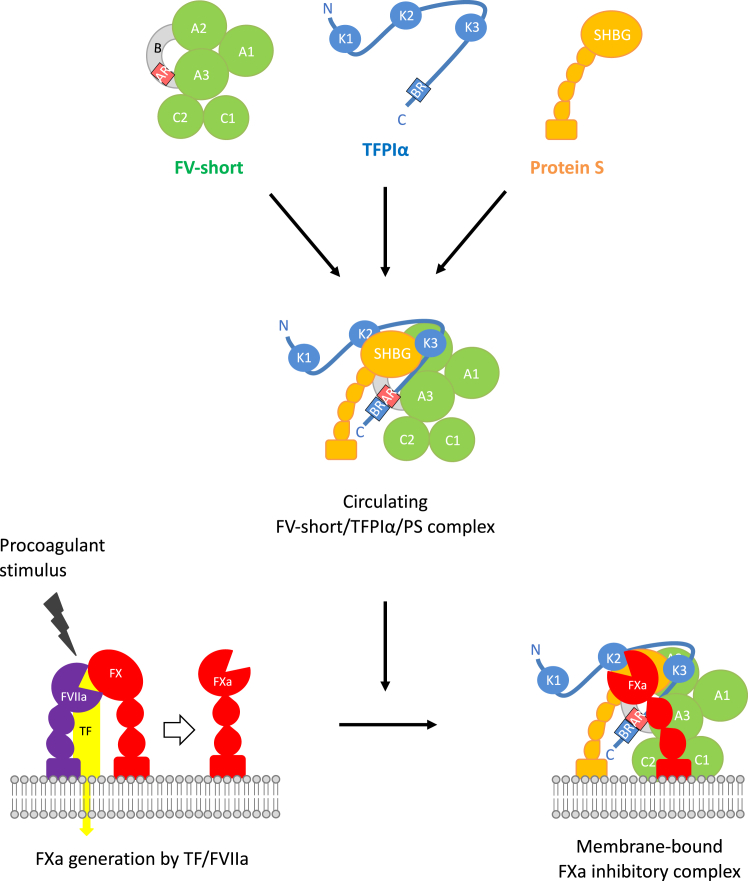
**Schematic representation of the assembly and function of the FV-short/TFPIα/protein S trimolecular complex.** FV-short, tissue factor pathway inhibitor α (TFPIα) and protein S (PS) assemble to form a tight trimolecular complex that circulates in plasma at low concentration (∼250 pM). This complex maintains TFPIα in the circulation and is primed to efficiently inhibit factor Xa (FXa) on the surface of negatively charged phospholipids. The presumed function of the complex would be to rapidly inhibit the first traces of FXa generated by the tissue factor/factor VIIa (TF/FVIIa) complex during initiation of coagulation, thereby regulating the transition to the propagation phase. *Source:* Figure adapted from Dahlbäck and Tran [44]. AR, acidic region; BR, basic region; K1, K2, K3, Kunitz-type domains of TFPIα; SHBG, sex hormone binding globulin domain of protein S.

Although these TFPIα-dependent anticoagulant functions were initially attributed to full-length FV, their main effector *in vivo* turned out to be FV-short [12,42], a low-abundance splicing isoform of FV that (thanks to its exposed AR) binds TFPIα with high affinity (Figure 1, inset). Intriguingly, immunoprecipitation experiments have indicated that FV-short is associated with both TFPIα and protein S in plasma, forming a trimolecular complex that is preassembled to optimally inhibit FXa (Figure 2) [44]. The assembly of this complex in solution relies on tight and cooperative interactions among all 3 proteins, involving the AR [12,21,45] and pre-AR [44,46] regions of FV-short, the C-terminus [21,44,47] and Kunitz-3 [47,48] domains of TFPIα, and the sex-hormone binding globulin domain of protein S [49,50]. Since the subnanomolar plasma concentrations of FV-short and TFPIα (both ∼250 pM) do not favor association, it has been proposed that protein S (∼150 nM in free form) may help to force all FV-short in complex with TFPIα, which is important to prevent the expression of the constitutive prothrombinase activity of free FV-short [46]. However, our understanding of the assembly and regulation of this trimolecular complex is still preliminary.

### 3.3 Similarities between the APC- and TFPIα-cofactor activities

Although the anticoagulant functions of FV as a cofactor of APC and TFPIα are carried out by different molecular forms, they share several common features. First, they both require the presence of protein S as an additional synergistic cofactor for APC [34] or TFPIα [41,42]. Moreover, they are both phospholipid-dependent, as demonstrated by the different APC- and TFPIα-cofactor activities of the FV1 and FV2 glycosylation isoforms [43,51], which bind to negatively charged phospholipids with different affinities [52]. In fact, the role of FV and protein S as synergistic anticoagulant cofactors would be to promote the binding of APC and TFPIα to phospholipids for efficient FVIIIa inactivation and FXa inhibition, respectively. Finally, both anticoagulant activities of FV are lost after the final FV activating cleavage at Arg^1545^, which separates the B domain from the light chain [37,[40], [41], [42], [43]], suggesting an important role of the C-terminal portion of the B domain in both activities.

## 4 FV and Bleeding

### 4.1 FV deficiency

Due to the essential procoagulant function of FVa in prothrombin activation, for which there is no physiological backup, the complete absence of FV is incompatible with life [18], while genetic and acquired FV deficiencies (FV:C <10%) are associated with mild-to-severe bleeding manifestations.

Congenital FV deficiency, also known as Owren’s parahemophilia, is an autosomal recessive disorder that affects ∼1:1,000,000 people in the general population and is caused by loss-of-function mutations in the *F5* gene [53,54]. Like for other coagulation factor deficiencies, there is considerable genetic heterogeneity, and ∼200 different *F5* mutations responsible for FV deficiency have been reported to date, including missense, nonsense, and splicing variants, as well as small indels and sporadic gene rearrangements [55]. Most mutations result in type I (quantitative) defects, characterized by a parallel reduction of FV antigen and activity, but a few type II (qualitative) defects have also been described, with normal antigen but reduced activity, mainly due to dissociation of the heavy and light chains after FV activation [[56], [57], [58], [59]].

**Figure 3:**
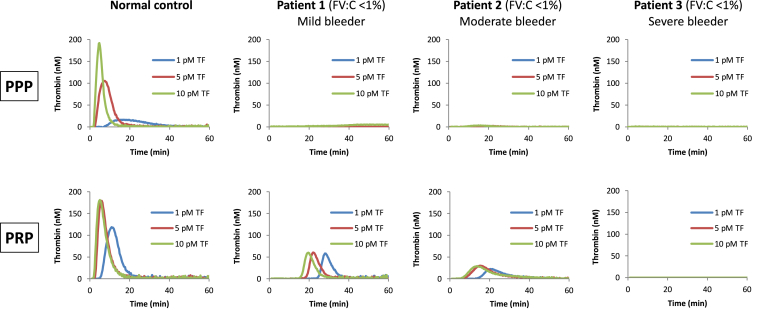
**Role of platelet FV in FV deficiency.** Comparison of thrombin generation in platelet-poor plasma (PPP, top) and platelet-rich-plasma (PRP, bottom) of a normal control and 3 patients with equally undetectable plasma FV (FV:C <1%) but different clinical phenotypes (mild, moderate, or severe). Thrombin generation was initiated with different concentrations of tissue factor (TF) in the presence of phospholipids (in PPP) or collagen (in PRP). While none of the patients’ PPP generated any thrombin, thrombin generation was measurable in the PRP of the mild and moderate bleeder (revealing the presence of functional platelet FV), but not in the PRP of the severe bleeder. The figure is based on data from different reports [[63], [64], [65]]. FV:C, factor V coagulant activity.

FV deficiency typically presents with mucosal and posttraumatic bleeding, whereas hemarthrosis and muscle hematomas are less common, and intracranial hemorrhages are generally rare [60]. While the plasma FV level is a rather poor predictor of the bleeding tendency, several studies have pointed at residual platelet FV as the main determinant of clinical severity [[61], [62], [63]]. In fact, several patients with undetectable plasma FV but mild or moderate bleeding symptoms proved to have small amounts of functional FV in their platelets [63,64], whereas at least one patient with life-threatening bleeding manifestations had undetectable FV in both plasma and platelets [65] (Figure 3). Platelet FV originates from endocytosis and subsequent modification of plasma FV by bone marrow megakaryocytes, which store the internalized FV in their α-granules and eventually release FV-loaded platelets [[66], [67], [68]]. How patients with undetectable plasma FV can build up FV in their platelets is unclear. However, since most patients’ mutations are compatible with some minimal FV secretion, megakaryocytes could accumulate this little plasma FV in their α-granules through continuous internalization over the course of their maturation (several days), protecting it from the rapid clearance that occurs in plasma. Moreover, platelet FV has enhanced procoagulant properties [67], and its targeted release at the site of injury further increases its hemostatic efficacy. Importantly, administration of fresh frozen plasma, which is currently the mainstay of prophylaxis and therapy in FV deficiency, has been shown to replenish the platelet FV pool, prolonging the hemostatic effect for several days after the plasma transfusion and well beyond the half-life of FV in plasma [69].

Since FV/FV-short is required to maintain TFPIα in circulation, FV deficiency is associated with markedly decreased plasma TFPIα levels [38]. As TFPIα directly antagonizes the procoagulant activity of FV [21,23], low TFPIα levels are beneficial to patients with severe FV deficiency, allowing traces of (platelet) FV to generate sufficient thrombin to guarantee minimal hemostasis [38,63].

The same protective mechanisms (integrity of the platelet FV pool [70] and low TFPIα levels [71]) apply to acquired FV deficiency caused by the development of anti-FV antibodies.

### 4.2 FV-short–related bleeding disorders

**Figure 4:**
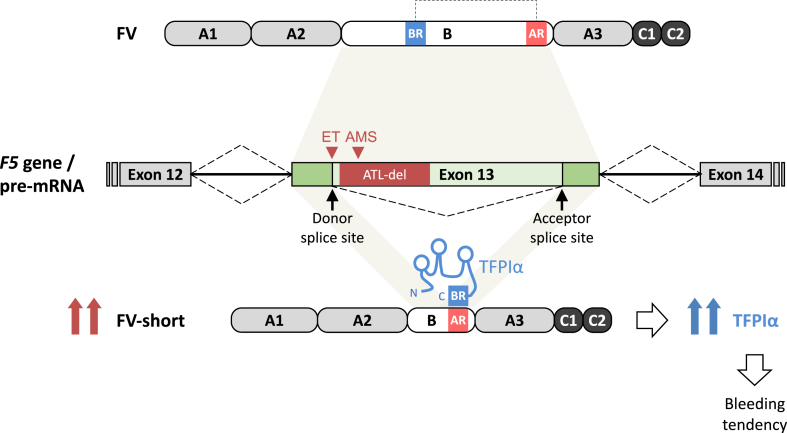
**FV-short splicing.** FV-short is generated by an alternative splicing event that removes an optional intron embedded within *F5* exon 13, causing the in-frame deletion of 702 amino acids (including the basic region [BR]) from the B domain of FV. This leaves the acidic region (AR) available for interaction with the BR of TFPIα. FV-short splicing normally occurs at very low levels but is greatly enhanced by the *F5*-East Texas (ET) and *F5*-Amsterdam (AMS) mutations, as well as by the *F5*-Atlanta deletion (ATL-del). These rare genetic defects result in considerable overexpression of FV-short (≥10-fold), which in turn can support more TFPIα in the circulation, causing a bleeding tendency. Figure adapted from Castoldi [76]. TFPIα, tissue factor pathway inhibitor α.

A novel mechanism of FV-related bleeding that has recently emerged is overexpression of FV-short, which normally represents only ∼1% of plasma FV (∼250 pM) [12]. FV-short was originally identified in a large family with the so-called East Texas bleeding disorder, an autosomal dominant condition manifesting as easy bruising, mucosal bleeding, and excessive post-traumatic bleeding, and characterized by prolonged PT and aPTT despite normal levels of all coagulation factors (including FV) [11]. The disorder was linked to an apparent missense mutation in the *F5* gene (*F5* c.2350A>G, Human Genome Variation Society nomenclature) [11], but the actual pathogenetic mechanism was elucidated only years later [12]. As it turned out, the *F5* c.2350A>G mutation enhances an alternative splicing event that generates a FV splicing isoform (FV-short) with a large in-frame deletion within the B domain (Figure 4). Since FV-short lacks the BR but retains the AR, it binds TFPIα with high affinity [45], stabilizing it in the circulation [12] and stimulating its FXa-inhibitory activity synergistically with protein S [42]. Therefore, the *F5*-East Texas mutation is associated with a major increase in TFPIα level (∼10-fold) and activity, resulting in delayed and decreased thrombin generation and a bleeding tendency [12].

As mentioned above, recent evidence suggests that virtually all FV-short circulates in a very tight complex with both protein S and TFPIα [44,46], which suppresses the constitutive prothrombinase activity of FV-short [12,45] while also protecting it from inactivation by APC [72]. This complex, where TFPIα is preassembled with both its cofactors, is primed to inhibit FXa, effectively blocking prothrombinase activity elicited by weak/accidental triggers [44,46] (Figure 2). In this way, the concentration of the FV-short/TFPIα/protein S complex sets a threshold for the generation of the first traces of thrombin at the onset of coagulation. This threshold is much higher in carriers of the *F5*-East Texas mutation than in normal individuals, which accounts for their bleeding diathesis [14]. Similarly, smaller interindividual differences in FV-short (complex) level in healthy individuals [12] may modulate this threshold (and hence the risk of bleeding or venous thrombosis) in the general population, but regrettably, no quantitative FV-short assay is yet available to test this hypothesis.

Since the elucidation of the East Texas bleeding disorder, three other cases of FV-short–related bleeding have been reported, all with similar patterns of post-traumatic bleeding but different genetic mechanisms of FV-short overexpression (Table 1 [11,12,[73], [74], [75]] and Figure 4). An apparently unrelated family from Indiana was found to segregate the same *F5* mutation identified in the East Texas bleeding disorder, which acts by strengthening the natural donor splice site for FV splicing [73]. Conversely, the *F5*-Amsterdam proband and her son carried a different point mutation that activates a cryptic donor splice site for FV-short splicing, generating a nonphysiological FV-short variant with a shorter B domain deletion but essentially the same functional properties as regular FV-short [74]. Finally, the severely affected *F5*-Atlanta patient presented exceptionally high FV-short and TFPIα levels, which were attributable to an 832-bp deletion within the FV-short-specific intron [75]. Since the donor and acceptor splice sites for FV-short splicing were normal in this patient, this finding strongly suggests that the *F5*-Atlanta deletion acts by removing one or more splicing silencers that normally inhibit FV-short splicing, revealing the existence and approximate location (if not yet the identity) of potent *cis*-acting regulators of FV-short splicing [75]. Consequently, this region of *F5* exon 13 may contain common and rare genetic determinants of FV-short expression (and hence TFPIα level and activity) that could modulate the clinical phenotype of hemorrhagic disorders, such as hemophilia, where TFPIα plays an important role [76].

**Table 1:** *F5* mutations increasing FV-short splicing.

Proband	Main bleeding symptoms	Mutation^a^	Mechanism of ↑ FV-short	TFPI levels	Reference
35**-**year-old male from Texas	Bruising, epistaxis, gum bleeding, bleeding after minor traumas	*F5* c.2350A>G (*F5-*East Texas)	Mutation strengthens natural donor splice site for FV-short splicing	Total: 5.3 nM Free: 1.25 nM	Kuang et al. 2001 [11] Vincent *et al.* 2013 [12]
20**-**year-old male (Caucasian)	Mucocutaneous bleeding Prolonged wound healing Posttraumatic bleeding	*F5* c.2350A>G (*F5-*East Texas)	Mutation strengthens natural donor splice site for FV-short splicing	Total: 5.7 nM^b^ Free: 2.9 nM^b^	Peterson et al. 2022 [73]
59-year-old female (Caucasian)	Bleeding after minor trauma Bleeding after surgery/delivery	*F5* c.2588C>G (*F5-*Amsterdam)	Mutation activates cryptic donor splice site for FV-short splicing	Total: 12.7 nM^b^ Free: 3.6 nM^b^	Cunha et al. 2015 [74]
43-year-old male (African American)	Umbilical stump bleeding Trauma-associated bleeding Recurrent muscular hematomas	*F5* c.2413_3244del (*F5-*Atlanta)	Deletion may remove negative regulator(s) of FV-short splicing	Total: 24.0 nM Free: 6.9 nM	Zimowski et al. 2021 [75]

a Annotation according to the Human Genome Variation Society (HGVS) nomenclature.

b Originally reported TFPI levels were converted to nM concentrations.

In the absence of a specific therapy, patients with FV-short–related bleeding disorders have been successfully managed with a variety of bypassing agents (factor eight inhibitor bypassing activity, prothrombin complex concentrate, and recombinant FVIIa), often in combination with antifibrinolytics [[73], [74], [75]]. Another treatment option that may be worth exploring is TFPI antagonists, as various anti-TFPI antibodies have shown efficacy in restoring *in vitro* thrombin generation when added directly to the plasma of these patients [12,[73], [74], [75]]. Alternatively, FV-short expression could be targeted directly using antisense-based splicing modulation strategies [77], although this approach is still hampered by our limited knowledge of the physiological regulation of FV-short splicing.

## 5 FV and Venous Thrombosis

### 5.1 APC resistance

APC resistance, originally identified in the context of familial thrombophilia, is a common condition characterized by poor sensitivity of plasma to the anticoagulant action of APC [9] and associated with an increased risk of venous thrombosis [78]. The main genetic cause of APC resistance is the FV Arg506Gln mutation (FV Leiden) [10], which is present in 5% of people of European descent and confers a 4- to 7-fold increased risk of venous thrombosis in the heterozygous state [79]. This mutation abolishes the kinetically favored APC-cleavage site at Arg^506^, predicting delayed FVa inactivation *via* slow cleavage at Arg^306^. In fact, FVa_Leiden_ is inactivated by APC alone ∼20-fold more slowly than normal FVa [25]. However, under more physiological conditions, ie, in the presence of protein S (which greatly stimulates cleavage at Arg^306^) and of FXa (which protects the Arg^506^ cleavage site in normal FVa), FVa_Leiden_ is inactivated at a similar rate as normal FVa [26]. Therefore, at least two other mechanisms have been proposed to explain the hypercoagulable state associated with FV Leiden. First, FV_Leiden_ lacks anticoagulant activity as a cofactor of APC and protein S in FVIIIa inactivation because this function requires APC-catalyzed cleavage of FV at Arg^506^ [36]. Second, it was recently reported that partially activated forms of FV_Leiden_ are less susceptible to prothrombinase inhibition by TFPIα than their normal counterparts [80], suggesting that the Arg506Gln amino acid substitution may weaken the FV-TFPIα interaction. If confirmed, this finding may also have consequences for the formation of the FV-short_Leiden_/TFPIα/protein S trimolecular complex and its ability to inhibit FXa.

**Figure 5:**
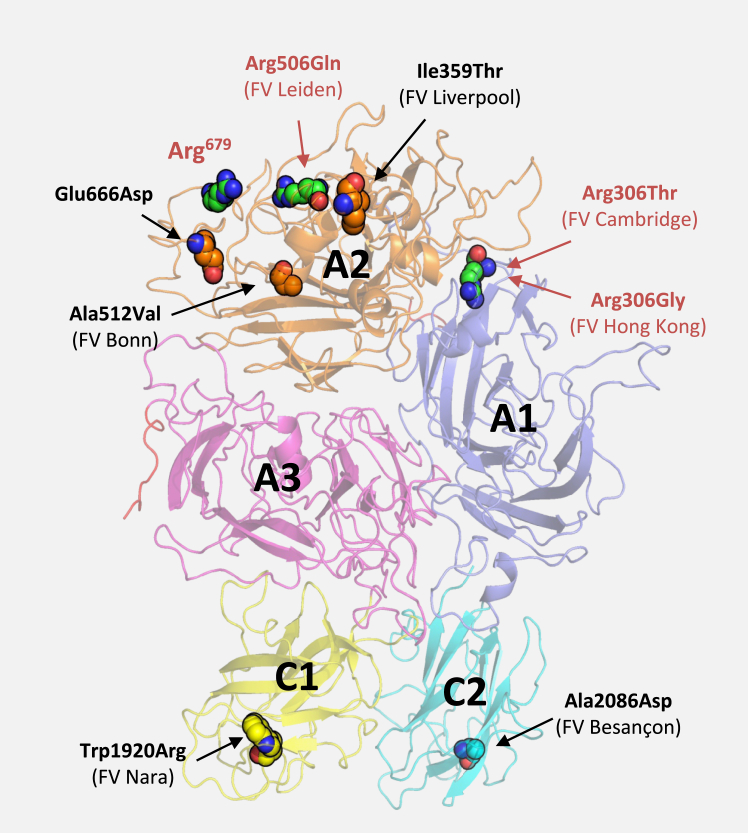
**Mapping of the FV variants associated with APC resistance on the structure of FV.** The structure of human FV (PDB ID 7KVE) [15] is shown as ribbon diagram, with the A and C domains highlighted in different colors. Residues marking the APC-cleavage sites (Arg^306^, Arg^506^, and Arg^679^) and other residues that were reported to be mutated in APC-resistant variants are emphasized in space-filling representation. FV variants affecting the APC-cleavage sites are indicated in red; all other APC-resistant variants are indicated in black. Figure: courtesy of Dr K. Wichapong.

After the discovery of FV Leiden, several other *F5* missense mutations have been reported to be associated with various degrees of APC resistance (see Table 2 and ref. [81] for a recent and more extensive review). Most of them reside in the heavy chain of FV, at or close to the main APC-cleavage sites (Figure 5), and their mechanisms of action can be rationalized in terms of reduced cleavage of FVa and/or FV by APC. For example, the mildly APC-resistant FV Cambridge (Arg306Thr) [82] and FV Hong Kong (Arg306Gly) [83] variants abolish the APC-cleavage site at Arg^306^, resulting in incomplete FVa inactivation (partially rescued by protein S) and slightly reduced APC-cofactor activity in FVIIIa inactivation [84]. Similar effects are associated with FV Liverpool (Ile359Thr) [85], which induces glycosylation of Asn^357^, thereby hindering APC-catalyzed cleavage of FVa at Arg^306^ and causing complete loss of APC-cofactor activity [86]. FV Bonn (Ala512Val), identified in several patients with venous thrombosis or (recurrent) abortion, interferes with proteolysis at the nearby Arg^506^ APC-cleavage site, causing moderate APC resistance through similar mechanisms as FV Leiden [87]. Interestingly, FV_Bonn_ is also more procoagulant in the absence of APC, possibly due to higher affinity for FXa [87], which might decrease its susceptibility to inhibition by TFPIα. Finally, the APC resistance associated with the FV Glu666Asp variant was tentatively attributed to impaired FVa inactivation due to inefficient cleavage at Arg^679^ [88].

**Table 2:** FV variants associated with APC resistance.

FV variant	Amino acid change	Ethnic distribution	Location	Mechanism	Reference
FV Leiden	Arg506Gln	Caucasians (5%)	Heavy chain (A2 domain)	Loss of the Arg^506^ APC-cleavage site	Bertina *et al.* 1994 [10]
FV Cambridge	Arg306Thr	Caucasians (sporadic)	Heavy chain (A1/A2 domain)	Loss of the Arg^306^ APC-cleavage site	Williamson *et al.* 1998 [82]
FV Hong Kong	Arg306Gly	Hong Kong Chinese (4.5%)	Heavy chain (A1/A2 domain)	Loss of the Arg^306^ APC-cleavage site	Chan *et al.* 1998 [83]
FV Liverpool	Ile359Thr	Caucasians (single family)	Heavy chain (A2 domain)	Gain of glycosylation site close to Arg^306^	Mumford *et al.* 2003 [85]
FV Bonn	Ala512Val	Caucasians (sporadic)	Heavy chain (A2 domain)	Amino acid change close to Arg^506^	Pezeshkpoor *et al.* 2016 [87]
-	Glu666Asp	Chinese (single family)	Heavy chain (A2 domain)	Amino acid change close to Arg^679^	Cai *et al.* 2010 [88]
FV Nara	Trp1920Arg	Japanese (single family)	Light chain (C1 domain)	Decreased binding affinity for phospholipids	Nogami *et al.* 2014 [89]
FV Besançon	Ala2086Asp	Single patient from Morocco	Light chain (C2 domain)	Decreased binding affinity for phospholipids	Castoldi *et al.* 2021 [90]

Amino acid numbering according to legacy nomenclature of Jenny et al. 1987 [7].

A completely different mechanism of APC resistance is exemplified by the FV Nara (Trp1920Arg) [89] and FV Besançon (Ala2086Asp) [90] variants, which map to the light chain of FV (Figure 5). As indicated by structural and functional analyses, these amino acid substitutions in the C1 and C2 domains interfere with FV/FVa binding to negatively charged phospholipids [[90], [91], [92]], affecting all procoagulant and anticoagulant functions of FV, but most prominently the APC-cofactor activity of FV [89,92] and the inactivation of FVa in the presence of protein S [[89], [90], [91], [92]]. The latter effect can be explained by the recent observation that FVa, in combination with protein S, greatly enhances APC binding to phospholipids, thereby promoting the assembly of its own FVa-inactivating complex [91]. In contrast to normal FVa, FVa_Nara_ proved unable to synergize with protein S to form this complex, resulting in impaired FVa inactivation, particularly in the presence of protein S and at suboptimal phospholipid concentrations [91]. Similar conclusions are likely to apply to the functionally comparable FV_Besançon_. These findings may account for the strong APC resistance and recurrent venous thrombosis events observed in the FV Nara and FV Besançon probands, both of whom were homozygous for the respective mutation [89,90]. An additional feature of both patients was their low FV level (FV:Ag 40% and 4%, respectively), predicting reduced anticoagulant activities of FV and low TFPIα levels.

These extreme examples illustrate how FV variants with a phospholipid-binding defect can induce a hypercoagulable state by creating an imbalance between the procoagulant and anticoagulant functions of FV. Interestingly, a common *F5* haplotype responsible for mild APC resistance (FV R2 [93]) has also been associated with a relative excess of a glycosylation isoform (FV1) that has reduced binding affinity for phospholipids [94]. Moreover, decreased phospholipid-binding capacity of FV was strongly associated with increased risk of venous thrombosis in a small case-control study from Japan [95].

### 5.2 FV levels and venous thrombosis risk

The fact that FV can express both procoagulant and anticoagulant activities makes it difficult to predict how variations in FV level will affect the hemostatic balance. The association between FV antigen levels and the risk of venous thrombosis has been recently investigated in the Multiple Environmental and Genetic Assessment of risk factors for venous thrombosis study, a large population-based case-control study comprising 2377 patients with a first episode of venous thromboembolism and 2939 controls [96]. Both low and high FV levels were associated with an increased risk of venous thrombosis, but the risk associated with high FV levels was largely attributable to concomitantly elevated FVIII levels. Differently, the risk associated with low FV levels increased after adjustment for FVIII levels and was even higher for unprovoked events, with an age- and sex-adjusted odds ratio of 2.64 (1.23-5.64) in subjects with FV:Ag levels <1^st^ percentile (57 U/dL) [96]. These findings indicate that—in contrast to severe FV deficiency—partial FV deficiency is associated with a hypercoagulable state. This apparently paradoxical conclusion might be explained by the different FV requirements for the expression of the procoagulant and anticoagulant activities of FV. As indicated by FV titrations of thrombin generation under different reaction conditions, the procoagulant activity of FV is already saturated at ∼10% FV [38], whereas the APC- and TFPIα-dependent anticoagulant activities increase throughout the physiological range of FV concentrations [43,97]. Therefore, at intermediate FV levels (20%-50%), the procoagulant activity of FV is already maximal, while its anticoagulant activities are still suboptimal, creating a procoagulant imbalance that can be further aggravated by the decreased TFPIα level [38].

This conclusion is corroborated by a recent study in patients with combined FV and FVIII deficiency, an autosomal recessive bleeding disorder characterized by low levels (typically 10%-20%) of both FV and FVIII [98]. Based on thrombin generation experiments in patients’ platelet-poor and platelet-rich plasma supplemented with FV or FVIII or collected after desmopressin infusion, it was shown that the bleeding tendency in these patients is due to the low FVIII level, whereas the low FV is protective rather than contributing to the hypocoagulable state [98]. In fact, the plasma and platelet FV levels in these patients were found to be sufficient for normal thrombin generation.

## 6 Conclusion

FV can take several molecular forms that express different procoagulant and anticoagulant activities and are differentially regulated by APC and TFPIα. As a result of this complexity, quantitative and qualitative alterations of FV can increase the risk of bleeding or venous thrombosis by a variety of mechanisms, as discussed in this review. Therefore, when evaluating the potential effects of *F5* gene variants on the hemostatic balance or when developing new treatments for FV-related disorders, all FV functions should be considered.

## 7 International Society on Thrombosis and Haemostasis Congress Report

Björn Dahlbäck gave a plenary lecture (PL 01.2) about his life-long career in FV research, with APC resistance and the East Texas bleeding disorder as his most seminal contributions. He also presented an original abstract illustrating the ability of FVa_int_ to function as a cofactor of TFPIα and protein S in the inhibition of FXa, just as FV-short, further underscoring the functional similarities between these forms of FV [99].

Enrico Di Cera presented a three-dimensional structure of FV-short obtained by cryogenic electron microscopy, which is now published [100]. In contrast to the previously published structure of FV, this structure includes the whole FV-short B domain, affording insights into the functionally important AR and pre-AR regions. It also accounts for the unique functional properties of FV-short (constitutive prothrombinase activity and high-affinity TFPIα binding) and can serve as a template to better understand the intramolecular interaction between the BR and AR of FV [100].

Finally, at the SSC session on Physiological Anticoagulants and Thrombophilia, Eriko Morishita presented a new FV variant, FV Kanazawa (Tyr1961Cys), associated with severe APC resistance in two Japanese siblings with recurrent venous thrombosis (SSC 01.4). Like FV Nara, FV Kanazawa maps to the C1 domain and is predicted to act through impaired phospholipid binding.

## 7 Future Directions

Following the elucidation of the East Texas bleeding disorder, recent years have witnessed a rapid accumulation of new and exciting observations on the interaction between TFPIα and various forms of FV, particularly FV-short. However, many questions remain unanswered and should be addressed in future studies.

First of all, it is still unclear whether platelet FV and FXa-cleaved FV (FVa_int_) are physiological targets of prothrombinase inhibition by (platelet) TFPIα [13,45], and—if so—whether these partially activated forms of FV can be recruited as anticoagulant cofactors of TFPIα/protein S in the inhibition of FXa, as suggested by Dahlbäck’s *in vitro* observations [99].

Moreover, little information is available on the assembly and regulation of the circulating FV-short/TFPIα/protein S complex. Although the interaction sites among the 3 proteins have been mapped to some extent [12,21,[44], [45], [46], [47], [48], [49], [50]], structural information and additional mutagenesis studies are required to better understand how the complex fits together. An important point is how complete association between FV-short and TFPIα is enforced *in vivo* and whether there are (pathological) conditions where the complex might be partially dissociated, as free FV-short is constitutively active [45] and potentially very procoagulant. Along the same lines, attention should be given to the possibility that the anti-TFPIα agents currently under development as bypassing agents for hemophilia may affect the stability of the complex.

To gain more insight into the physiological role of the FV-short/TFPIα/protein S complex, an assay is needed to measure its plasma concentration and to determine its association with the risk of bleeding and venous thrombosis in population studies. While the development of a FV-short ELISA has proved particularly challenging, the structure and function of the complex may suggest alternative quantification strategies.

Finally, another area of interest is the regulation of FV-short splicing. A better understanding of the *cis*-acting regulatory elements and corresponding *trans-*acting factors that promote or suppress FV-short splicing may point out new therapeutic targets for the downregulation of FV-short expression in TFPIα-dependent bleeding disorders.

## References

[1] Mast A.E. (2016). Tissue factor pathway inhibitor: multiple anticoagulant activities for a single protein. Arterioscler Thromb Vasc Biol.

[2] Dahlbäck B., Villoutreix B.O. (2005). The anticoagulant protein C pathway. FEBS Lett.

[3] Dahlbäck B. (2016). Pro- and anticoagulant properties of factor V in pathogenesis of thrombosis and bleeding disorders. Int J Lab Hematol.

[4] Owren P.A. (1947). Parahaemophilia; haemorrhagic diathesis due to absence of a previously unknown clotting factor. Lancet.

[5] Stormorken H. (2003). The discovery of factor V: a tricky clotting factor. J Thromb Haemost.

[6] Rosing J., Tans G., Govers-Riemslag J.W., Zwaal R.F., Hemker H.C. (1980). The role of phospholipids and factor Va in the prothrombinase complex. J Biol Chem.

[7] Jenny R.J., Pittman D.D., Toole J.J., Kriz R.W., Aldape R.A., Hewick R.M. (1987). Complete cDNA and derived amino acid sequence of human factor V. Proc Natl Acad Sci U S A.

[8] Cripe L.D., Moore K.D., Kane W.H. (1992). Structure of the gene for human coagulation factor V. Biochemistry.

[9] Dahlbäck B., Carlsson M., Svensson P.J. (1993). Familial thrombophilia due to a previously unrecognized mechanism characterized by poor anticoagulant response to activated protein C: prediction of a cofactor to activated protein C.. Proc Natl Acad Sci U S A.

[10] Bertina R.M., Koeleman B.P., Koster T., Rosendaal F.R., Dirven R.J., de Ronde H. (1994). Mutation in blood coagulation factor V associated with resistance to activated protein C. Nature.

[11] Kuang S.Q., Hasham S., Phillips M.D., Wolf D., Wan Y., Thiagarajan P. (2001). Characterization of a novel autosomal dominant bleeding disorder in a large kindred from east Texas. Blood.

[12] Vincent L.M., Tran S., Livaja R., Bensend T.A., Milewicz D.M., Dahlbäck B. (2013). Coagulation factor V(A2440G) causes east Texas bleeding disorder via TFPIalpha. J Clin Invest.

[13] Camire R.M. (2016). Rethinking events in the haemostatic process: role of factor V and TFPI. Haemophilia.

[14] Dahlbäck B. (2023). Natural anticoagulant discovery, the gift that keeps on giving: finding FV-Short. J Thromb Haemost.

[15] Ruben E.A., Rau M.J., Fitzpatrick J.A.J., Di Cera E. (2021). Cryo-EM structures of human coagulation factors V and Va. Blood.

[16] Krishnaswamy S., Church W.R., Nesheim M.E., Mann K.G. (1987). Activation of human prothrombin by human prothrombinase. Influence of factor Va on the reaction mechanism. J Biol Chem.

[17] Ruben E.A., Summers B., Rau M.J., Fitzpatrick J.A.J., Di Cera E. (2022). Cryo-EM structure of the prothrombin-prothrombinase complex. Blood.

[18] Cui J., O'Shea K.S., Purkayastha A., Saunders T.L., Ginsburg D. (1996). Fatal haemorrhage and incomplete block to embryogenesis in mice lacking coagulation factor V. Nature.

[19] Monkovic D.D., Tracy P.B. (1990). Activation of human factor V by factor Xa and thrombin. Biochemistry.

[20] Bos M.H., Camire R.M. (2012). A bipartite autoinhibitory region within the B-domain suppresses function in factor V. J Biol Chem.

[21] Wood J.P., Bunce M.W., Maroney S.A., Tracy P.B., Camire R.M., Mast A.E. (2013). Tissue factor pathway inhibitor-alpha inhibits prothrombinase during the initiation of blood coagulation. Proc Natl Acad Sci U S A.

[22] Wood J.P., Petersen H.H., Yu B., Wu X., Hilden I., Mast A.E. (2017). TFPIalpha interacts with FVa and FXa to inhibit prothrombinase during the initiation of coagulation. Blood Adv.

[23] van Doorn P., Rosing J., Wielders S.J., Hackeng T.M., Castoldi E. (2017). The C-terminus of tissue factor pathway inhibitor-alpha inhibits factor V activation by protecting the Arg(1545) cleavage site. J Thromb Haemost.

[24] Kalafatis M., Rand M.D., Mann K.G. (1994). The mechanism of inactivation of human factor V and human factor Va by activated protein C. J Biol Chem.

[25] Nicolaes G.A., Tans G., Thomassen M.C., Hemker H.C., Pabinger I., Váradi K. (1995). Peptide bond cleavages and loss of functional activity during inactivation of factor Va and factor VaR506Q by activated protein C. J Biol Chem.

[26] Rosing J., Hoekema L., Nicolaes G.A., Thomassen M.C., Hemker H.C., Váradi K. (1995). Effects of protein S and factor Xa on peptide bond cleavages during inactivation of factor Va and factor VaR506Q by activated protein C. J Biol Chem.

[27] Norstrøm E.A., Steen M., Tran S., Dahlbäck B. (2003). Importance of protein S and phospholipid for activated protein C-mediated cleavages in factor Va. J Biol Chem.

[28] Norstrøm E.A., Tran S., Steen M., Dahlbäck B. (2006). Effects of factor Xa and protein S on the individual activated protein C-mediated cleavages of coagulation factor Va. J Biol Chem.

[29] Kim P.Y., Nesheim M.E. (2010). Down regulation of prothrombinase by activated protein C during prothrombin activation. Thromb Haemost.

[30] Tran S., Norstrøm E., Dahlbäck B. (2008). Effects of prothrombin on the individual activated protein C-mediated cleavages of coagulation factor Va. J Biol Chem.

[31] Yegneswaran S., Nguyen P.M., Gale A.J., Griffin J.H. (2009). Prothrombin amino terminal region helps protect coagulation factor Va from proteolytic inactivation by activated protein C.. Thromb Haemost.

[32] Li X., Song X., Mahmood D.F.D., Sim M.M.S., Bidarian S.J., Wood J.P. (2023). Activated protein C, protein S, and tissue factor pathway inhibitor cooperate to inhibit thrombin activation. Thromb Res.

[33] Dahlbäck B., Hildebrand B. (1994). Inherited resistance to activated protein C is corrected by anticoagulant cofactor activity found to be a property of factor V. Proc Natl Acad Sci U S A.

[34] Shen L., Dahlbäck B. (1994). Factor V and protein S as synergistic cofactors to activated protein C in degradation of factor VIIIa. J Biol Chem.

[35] Gale A.J., Cramer T.J., Rozenshteyn D., Cruz J.R. (2008). Detailed mechanisms of the inactivation of factor VIIIa by activated protein C in the presence of its cofactors, protein S and factor V.. J Biol Chem.

[36] Thorelli E., Kaufman R.J., Dahlbäck B. (1999). Cleavage of factor V at Arg 506 by activated protein C and the expression of anticoagulant activity of factor V. Blood.

[37] Thorelli E., Kaufman R.J., Dahlbäck B. (1998). The C-terminal region of the factor V B-domain is crucial for the anticoagulant activity of factor V. J Biol Chem.

[38] Duckers C., Simioni P., Spiezia L., Radu C., Gavasso S., Rosing J. (2008). Low plasma levels of tissue factor pathway inhibitor in patients with congenital factor V deficiency. Blood.

[39] Hackeng T.M., Seré K.M., Tans G., Rosing J. (2006). Protein S stimulates inhibition of the tissue factor pathway by tissue factor pathway inhibitor. Proc Natl Acad Sci U S A.

[40] Peraramelli S., Thomassen S., Heinzmann A., Hackeng T.M., Hartmann R., Scheiflinger F. (2016). Role of exosite binding modulators in the inhibition of Fxa by TFPI. Thromb Haemost.

[41] Santamaria S., Reglińska-Matveyev N., Gierula M., Camire R.M., Crawley J.T.B., Lane D.A. (2017). Factor V has an anticoagulant cofactor activity that targets the early phase of coagulation. J Biol Chem.

[42] Dahlbäck B., Guo L.J., Livaja-Koshiar R., Tran S. (2018). Factor V-short and protein S as synergistic tissue factor pathway inhibitor (TFPIalpha) cofactors. Res Pract Thromb Haemost.

[43] van Doorn P., Rosing J., Duckers C., Hackeng T.M., Simioni P., Castoldi E. (2018). Factor V has anticoagulant activity in plasma in the presence of TFPIalpha: difference between FV1 and FV2. Thromb Haemost.

[44] Dahlbäck B., Tran S. (2022). A hydrophobic patch (PLVIVG; 1481-1486) in the B-domain of factor V-short is crucial for its synergistic TFPIalpha-cofactor activity with protein S and for the formation of the FXa-inhibitory complex comprising FV-short, TFPIalpha, and protein S.. J Thromb Haemost.

[45] Petrillo T., Ayombil F. (2021). Van't Veer C, Camire RM. Regulation of factor V and factor V-short by TFPIalpha: Relationship between B-domain proteolysis and binding. J Biol Chem.

[46] Dahlbäck B., Tran S. (2022). The preAR2 region (1458-1492) in factor V-Short is crucial for the synergistic TFPIalpha-cofactor activity with protein S and the assembly of a trimolecular factor Xa-inhibitory complex comprising FV-Short, protein S, and TFPIalpha. J Thromb Haemost.

[47] Gierula M., Noakes V.M., Salles C., Crawley J.T.B., Ahnstrom J. (2023). The TFPIalpha C-terminal tail is essential for TFPIalpha-FV-short-protein S complex formation and synergistic enhancement of TFPIalpha. J Thromb Haemost.

[48] Ndonwi M., Tuley E.A., Broze G.J. (2010). The Kunitz-3 domain of TFPI-alpha is required for protein S-dependent enhancement of factor Xa inhibition. Blood.

[49] Reglińska-Matveyev N., Andersson H.M., Rezende S.M., Dahlback B., Crawley J.T., Lane D.A. (2014). TFPI cofactor function of protein S: essential role of the protein S SHBG-like domain. Blood.

[50] Teraz-Orosz A., Gierula M., Petri A., Jones D., Keniyopoullos R., Folgado P.B. (2022). Laminin G1 residues of protein S mediate its TFPI cofactor function and are competitively regulated by C4BP. Blood Adv.

[51] Govers-Riemslag J.W., Castoldi E., Nicolaes G.A., Tans G., Rosing J. (2002). Reduced factor V concentration and altered FV1/FV2 ratio do not fully explain R2-associated APC-resistance. Thromb Haemost.

[52] Rosing J., Bakker H.M., Thomassen M.C., Hemker H.C., Tans G. (1993). Characterization of two forms of human factor Va with different cofactor activities. J Biol Chem.

[53] Asselta R., Peyvandi F. (2009). Factor V deficiency. Semin Thromb Hemost.

[54] Tabibian S., Shiravand Y., Shams M., Safa M., Gholami M.S., Heydari F. (2019). A comprehensive overview of coagulation factor V and congenital factor V deficiency. Semin Thromb Hemost.

[55] Efthymiou C., Print E.H.T., Simmons A., Perkins S.J. (2023). Analysis of 363 genetic variants in F5 via an interactive web database reveals new insights into FV deficiency and FV leiden. TH Open.

[56] Murray J.M., Rand M.D., Egan J.O., Murphy S., Kim H.C., Mann K.G. (1995). Factor VNew Brunswick: Ala221-to-Val substitution results in reduced cofactor activity. Blood.

[57] Steen M., Miteva M., Villoutreix B.O., Yamazaki T., Dahlbäck B. (2003). Factor V New Brunswick: Ala221Val associated with FV deficiency reproduced in vitro and functionally characterized. Blood.

[58] Calzavarini S., Villoutreix B.O., Lunghi B., Livaja R., Bernardi F., Dahlbäck B. (2013). Molecular basis of coagulation factor V deficiency caused by the R1698W inter-domain mutation. Thromb Haemost.

[59] Liu H.C., Shen M.C., Eng H.L., Wang C.H., Lin T.M. (2014). Asp68His mutation in the A1 domain of human factor V causes impaired secretion and ineffective translocation. Haemophilia.

[60] Lak M., Sharifian R., Peyvandi F., Mannucci P.M. (1998). Symptoms of inherited factor V deficiency in 35 Iranian patients. Br J Haematol.

[61] Miletich J.P., Majerus D.W., Majerus P.W. (1978). Patients with congenital factor V deficiency have decreased factor Xa binding sites on their platelets. J Clin Invest.

[62] Shinozawa K., Amano K., Suzuki T., Tanaka A., Iijima K., Takahashi H. (2007). Molecular characterization of 3 factor V mutations, R2174L, V1813M, and a 5-bp deletion, that cause factor V deficiency. Int J Hematol.

[63] Duckers C., Simioni P., Spiezia L., Radu C., Dabrilli P., Gavasso S. (2010). Residual platelet factor V ensures thrombin generation in patients with severe congenital factor V deficiency and mild bleeding symptoms. Blood.

[64] Todaro A.M., Radu C.M., Ciccone M., Toffanin S., Serino M.L., Campello E. (2023). In vitro and ex vivo rescue of a nonsense mutation responsible for severe coagulation factor V deficiency. J Thromb Haemost.

[65] Castoldi E., Duckers C., Radu C., Spiezia L., Rossetto V., Tagariello G. (2011). Homozygous F5 deep-intronic splicing mutation resulting in severe factor V deficiency and undetectable thrombin generation in platelet-rich plasma. J Thromb Haemost.

[66] Camire R.M., Pollak E.S., Kaushansky K., Tracy P.B. (1998). Secretable human platelet-derived factor V originates from the plasma pool. Blood.

[67] Gould W.R., Silveira J.R., Tracy P.B. (2004). Unique in vivo modifications of coagulation factor V produce a physically and functionally distinct platelet-derived cofactor: characterization of purified platelet-derived factor V/Va. J Biol Chem.

[68] Gertz J.M., Bouchard B.A. (2015). Mechanisms regulating acquisition of platelet-derived factor V/Va by megakaryocytes. J Cell Biochem.

[69] Bouchard B.A., Chapin J., Brummel-Ziedins K.E., Durda P., Key N.S., Tracy P.B. (2015). Platelets and platelet-derived factor Va confer hemostatic competence in complete factor V deficiency. Blood.

[70] Nesheim M.E., Nichols W.L., Cole T.L., Houston J.G., Schenk R.B., Mann K.G. (1986). Isolation and study of an acquired inhibitor of human coagulation factor V. J Clin Invest.

[71] De Maertelaere E., Castoldi E., Van Haute I., Deeren D., Devreese K.M. (2018). The interaction of factor V and tissue factor pathway inhibitor in a myeloma patient with acquired factor V deficiency. Haemophilia.

[72] Ayombil F., Petrillo T., Kim H., Camire R.M. (2022). Regulation of factor V by the anticoagulant protease activated protein C: Influence of the B-domain and TFPIalpha. J Biol Chem.

[73] Peterson J.A., Gupta S., Martinez N.D., Hardesty B., Maroney S.A., Mast A.E. (2022). Factor V east Texas variant causes bleeding in a three-generation family. J Thromb Haemost.

[74] Cunha M.L., Bakhtiari K., Peter J., Marquart J.A., Meijers J.C., Middeldorp S. (2015). A novel mutation in the F5 gene (factor V Amsterdam) associated with bleeding independent of factor V procoagulant function. Blood.

[75] Zimowski K.L., Petrillo T., Ho M.D., Wechsler J., Shields J.E., Denning G. (2021). F5-Atlanta: a novel mutation in F5 associated with enhanced East Texas splicing and FV-short production. J Thromb Haemost.

[76] Castoldi E. (2021). F5-Atlanta: factor V-short strikes again. J Thromb Haemost.

[77] Todaro A.M., Hackeng T.M., Castoldi E. (2021). Antisense-mediated down-regulation of factor V-short splicing in a liver cell line model. Appl Sci.

[78] Svensson P.J., Dahlbäck B. (1994). Resistance to activated protein C as a basis for venous thrombosis. N Engl J Med.

[79] Bezemer I.D., Bare L.A., Doggen C.J., Arellano A.R., Tong C., Rowland C.M. (2008). Gene variants associated with deep vein thrombosis. JAMA.

[80] Wood J.P., Baumann Kreuziger L.M., Ellery P.E.R., Maroney S.A., Mast A.E. (2017). Reduced prothrombinase inhibition by tissue factor pathway inhibitor contributes to the factor V leiden hypercoagulable state. Blood Adv.

[81] Moore G.W., Castoldi E., Teruya J., Morishita E., Adcock D.M. (2023). Factor V Leiden-independent activated protein C resistance: Communication from the plasma coagulation inhibitors subcommittee of the International Society on Thrombosis and Haemostasis Scientific and Standardisation Committee. J Thromb Haemost.

[82] Williamson D., Brown K., Luddington R., Baglin C., Baglin T. (1998). Factor V Cambridge: a new mutation (Arg306-->Thr) associated with resistance to activated protein C. Blood.

[83] Chan W.P., Lee C.K., Kwong Y.L., Lam C.K., Liang R. (1998). A novel mutation of Arg306 of factor V gene in Hong Kong Chinese. Blood.

[84] Norstrøm E., Thorelli E., Dahlbäck B. (2002). Functional characterization of recombinant FV Hong Kong and FV Cambridge. Blood.

[85] Mumford A.D., McVey J.H., Morse C.V., Gomez K., Steen M., Norstrøm E.A. (2003). Factor V I359T: a novel mutation associated with thrombosis and resistance to activated protein C. Br J Haematol.

[86] Steen M., Norstrøm E.A., Tholander A.L., Bolton-Maggs P.H., Mumford A., McVey J.H. (2004). Functional characterization of factor V-Ile359Thr: a novel mutation associated with thrombosis. Blood.

[87] Pezeshkpoor B., Castoldi E., Mahler A., Hanel D., Müller J., Hamedani N.S. (2016). Identification and functional characterization of a novel F5 mutation (Ala512Val, FV Bonn) associated with activated protein C resistance. J Thromb Haemost.

[88] Cai H., Hua B., Fan L., Wang Q., Wang S., Zhao Y. (2010). A novel mutation (g2172-->c) in the factor V gene in a Chinese family with hereditary activated protein C resistance. Thromb Res.

[89] Nogami K., Shinozawa K., Ogiwara K., Matsumoto T., Amano K., Fukutake K. (2014). Novel FV mutation (W1920R, FVNara) associated with serious deep vein thrombosis and more potent APC resistance relative to FVLeiden. Blood.

[90] Castoldi E., Hézard N., Mourey G., Wichapong K., Poggi M., Ibrahim-Kosta M. (2021). Severe thrombophilia in a factor V-deficient patient homozygous for the Ala2086Asp mutation (FV Besançon). J Thromb Haemost.

[91] Gierula M., Salles C., Santamaria S., Teraz-Orosz A., Crawley J.T.B., Lane D.A. (2019). The roles of factor Va and protein S in formation of the activated protein C/protein S/factor Va inactivation complex. J Thromb Haemost.

[92] Shimonishi N., Ogiwara K., Yoshida J., Horie K., Nakajima Y., Furukawa S. (2023). Impaired factor V-related anticoagulant mechanisms and deep vein thrombosis associated with A2086D and W1920R mutations. Blood Adv.

[93] Bernardi F., Faioni E.M., Castoldi E., Lunghi B., Castaman G., Sacchi E. (1997). A factor V genetic component differing from factor V R506Q contributes to the activated protein C resistance phenotype. Blood.

[94] Hoekema L., Castoldi E., Tans G., Girelli D., Gemmati D., Bernardi F. (2001). Functional properties of factor V and factor Va encoded by the R2-gene. Thromb Haemost.

[95] Suehisa E., Kawasaki T., Toku M., Hidaka Y. (2010). Low level of factor V is associated with development of deep-vein thrombosis in Japanese patients. Thromb Res.

[96] Rietveld I.M., Bos M.H.A., Lijfering W.M., Li-Gao R., Rosendaal F.R., Reitsma P.H. (2018). Factor V levels and risk of venous thrombosis: the MEGA case-control study. Res Pract Thromb Haemost.

[97] Castoldi E., Brugge J.M., Nicolaes G.A., Girelli D., Tans G., Rosing J. (2004). Impaired APC cofactor activity of factor V plays a major role in the APC resistance associated with the factor V Leiden (R506Q) and R2 (H1299R) mutations. Blood.

[98] Shao Y., Wu W., Xu G., Wang X., Ding Q. (2019). Low factor V level ameliorates bleeding diathesis in patients with combined deficiency of factor V and factor VIII.. Blood.

[99] Dahlbäck B., Tran S. (2023). Anticoagulant properties of Factor V activation intermediate (FVai) functioning as synergistic TFPI-cofactor with protein S [abstract]. Res Pract Thromb Haemost.

[100] Mohammed B.M., Pelc L.A., Rau M.J., Di Cera E. (2023). Cryo-EM structure of coagulation factor V short. Blood.

